# The role of elastography in determining the risk of malignant thyroid nodules in children

**DOI:** 10.3389/fendo.2024.1461031

**Published:** 2024-11-15

**Authors:** Aleksandra Kiszka-Wiłkojć, Anna Taczanowska-Niemczuk, Dominika Januś, Marcin Maślanka, Joanna Godlewska, Monika Kujdowicz, Michał Wiłkojć, Wojciech Górecki

**Affiliations:** ^1^ Department of Pediatric Surgery, Institute of Pediatrics, Jagiellonian University Medical College, Krakow, Poland; ^2^ Department of Pediatric Surgery, University Children’s Hospital of Krakow, Krakow, Poland; ^3^ Department of Pediatric and Adolescent Endocrinology, Chair of Pediatrics, Institute of Pediatrics, Jagiellonian University Medical College, Krakow, Poland; ^4^ Department of Pediatric and Adolescent Endocrinology, University Children’s Hospital in Krakow, Krakow, Poland; ^5^ Department of Pathology, University Children’s Hospital of Krakow, Krakow, Poland; ^6^ Department of Pathomorphology, Jagiellonian University Medical College, Krakow, Poland; ^7^ Department of General, Oncological, Metabolic and Thoracic Surgery, Military Institute of Medicine, National Research Institute, Warsaw, Poland

**Keywords:** ultrasonography, thyroid nodule, strain elastography, EU-TIRADS, thyroid cancer

## Abstract

**Introduction:**

Ultrasonography is fundamental method of diagnosing focal thyroid lesions. The additional element of ultrasound examination is Strain Elastography which allows for determining the degree of elasticity of the nodule while comparing it to the surrounding thyroid parenchyma. Pediatric thyroid nodules have a higher malignancy risk than in adults, warranting the consideration of fine-needle aspiration biopsy (FNAB) in children.

**Material and methods:**

A prospective data analysis of children with focal thyroid lesions treated from 2021 to 2022 was performed. The patients underwent ultrasound and elastography examinations to obtain the Strain Ratio (SR) of the nodules and were qualified for FNAB. SR was determined by the windowing method of relative strains in a semi-quantitative assessment. The FNAB score was determined on the Bethesda scale and the histological examination of the thyroid nodule was performed. The SR values were stratified in three groups: I - thyroid cancer, II - low-risk thyroid tumors, and III - benign lesion. The Kruskal-Wallis test was used to find the relation between the value of SR elastography and the malignancy, with the p value < 0.05 considered significant. The data were analyzed using the multiple comparisons test.

**Results:**

The 123 FNABs were performed in 100 patients. The final analysis included 119 nodules in 96 patients. In 19 cases, the nodule was malignant, in 5 cases they were low-risk tumors, and in 95 - benign lesions. A difference of the SR value between groups in the pairs of malignant and benign tumors, and malignant and low-risk tumors was revealed. Since no statistically significant difference in the level of elastography was found between benign and low-risk tumors, both groups were combined and formed a group of benign tumors. For the combined groups, the Man-Whitney test was performed, confirming that there was a statistically significant difference between the groups of malignant and benign tumors in the value of SR elastography. The cut-off point for SR for malignant tumors was >3.

**Conclusions:**

The SR index of elastography is significantly higher in malignant nodules. and might be used to select changes with an increased risk of malignancy in thyroid ultrasound of children.

## 1 Introduction

Ultrasonography (USG) of the thyroid gland is a basic diagnostic tool for identifying changes that may require further invasive diagnostic and surgical management. The distribution of ultrasound features observed in cases of thyroid nodule evaluation, indicating the risk of malignancy, is widely known. There are many scales that describe the ultrasonographic features of a thyroid nodule. The European Thyroid Imaging Reporting and Data System (TI-RADS) scale was developed to accurately determine the indications for further diagnostic management in 2017 in adults (1). The most suspicious features of nodule malignancy in ultrasound are hypoechogenicity, a “taller-than-wide” shape in the transverse plane, irregular margins, and calcification. The above features of the nodule allow for the qualification of the lesion for fine-needle aspiration biopsy (FNAB) (1). According to the American Thyroid Association, suspicious ultrasound features indicating thyroid malignancy are very similar to these reported by EU-TI-RADS and also include the assessment of the margins and composition; furthermore, a number of points are assigned to each feature, and the final risk is evaluated as the sum of the points (2).

In the pediatric population, scales such as TI-RADS, American Thyroid Association (ATA), or American College of Radiology (ACR) TI-RADS have moderate diagnostic performance in thyroid nodule evaluation (3).

However, when we use them as an additional tool in combination with clinical history (genetic predisposition, previous exposure to radiation, age, male gender, and autoimmune thyroid disease), EU-TI-RADS may be an adequate and reproducible method to estimate the suspicion of malignancy in pediatric patients (4). A suspicious thyroid nodule is one that exhibits the following features on ultrasound examination: hypoechogenicity, a hyperechogenic “border” between a nodule and thyroid parenchyma, poorly defined margins, irregular shape, microcalcifications, solid composition, and increased growth potential observed during ultrasound follow-up (5). In a recent study, Ortega et al. suggested that TI-RADS can risk-stratify pediatric thyroid nodules; however, the system requires modifications to reduce the missed malignancy rate in pediatric thyroid nodules by lowering size thresholds for FNAB in children (6).

Based on the FNAB results, patients are qualified for surgical treatment. Following thyroid resection along with the nodule, the final histological result of the nodule is obtained.

The follicular-derived tumors are divided into three categories: benign (thyroid nodular disease and adenomas), low risk [non-invasive follicular thyroid neoplasm with papillary-like nuclear features (NIFTP), follicular tumors of uncertain malignant potential (FT-UMP), well-differentiated tumor of uncertain malignant potential (WDT-UMP), and hyalinizing trabecular tumor], and malignant [papillary thyroid carcinoma (PTC), follicular, medullary and anaplastic thyroid carcinoma, and oxyphilic carcinoma] (7, 8). The elastography is not a standard method in decision-making whether to perform the FNA biopsy, but it may help with its evaluation. The other method of thyroid nodule evaluation is infrared Raman spectroscopy (9, 10).

Thyroid nodules are less common in children as compared to adults (0.2%–5% and 19%–67%, respectively) (11, 12). Pediatric patients tend to have a higher malignancy rate than adults (22%–26% and 5%–10%, respectively) (13–15). The cytological evaluation with the Bethesda scale shows that 28% of thyroid nodules with BIII and 58% with BIV are malignant in histology (16). The malignancy rate is higher in children than in adults for the analogical Bethesda categories. In adults, the category BIII is associated with a 22% risk of thyroid cancer (the range 13%–30%), and in the BIV, the risk is 30% (23%–34%) (17).

The Polish guidelines for the diagnosis and treatment of thyroid cancer in children are based on the American Thyroid Association guidelines. The category BII denotes a benign change that should be observed in pediatrics, while BIII and BIV require surgical treatment (the thyroid lobectomy). Patients with BV and BVI categories are subjected to total thyroidectomy. Adult patients with BIII undergo observation and should have a repeated biopsy after 3–6 months (11, 13, 18, 19).

We present the attempts at the verification of focal thyroid changes in children by the addition of an option of the standard ultrasound assessment, which is elastography.

Elastography is a technique that has been developed to provide images and evaluate tissue stiffness. Palpation is a technique in which the clinician uses his hands to test the patient’s body for abnormalities and tumors. Palpation has been used since ancient times. Elastography can detect the stiffness of deeper and smaller lesions that are inaccessible to palpation. There are two main types of elastography: strain elastography (developed as the first method) and shear wave elastography. The development of stain elastography was reported by Jonathan Ophir in 1991 as a quasi-static technique in *Ultrasonic Imaging* (20).

## 2 Methods

Elastography works by applying external or internal compression (e.g., through breathing or vascular pulsing) to tissue using an ultrasound transducer. Strain images are created by applying minimal repetitive pressure and measuring tissue displacement through methods like RF echo correlation-based tracking, Doppler processing, or combined techniques. Displacement between RF echo frames is used to calculate strain, with stiffer regions showing less strain. The strain ratio quantifies static elastograms by comparing mean strain in lesion and reference areas (21, 22). Higher strain ratio (SR) values indicate a higher risk of malignancy (23).

The strain ratio method of quantifying static elastogram is as follows:


$$Strain\;Ratio=SR=\frac{Mean\;strain\;B\;(reference\;area)}{Mean\;strain\;A\;(lesion\;area)}$$

For the region of interest (ROI), A is the lesion area and B is the reference area/healthy tissue: thyroid tissue, muscles, and fat tissue.

Shear wave elastography, unlike strain imaging, uses dynamic stress to generate shear waves and measure tissue elasticity. Both techniques are used to assess thyroid nodules, with elasticity scored on scales like Tsukuba or Asteria, where higher scores suggest greater malignancy risk (19, 23).

This prospective study was performed between June 2021 and December 2022 in the Department of Pediatric Surgery University Children’s Hospital of Krakow, Poland. The consent of the Bioethics Committee of the Jagiellonian University in Krakow No. 1072.6120.288.2021 was obtained. Written informed consent for FNAB and elastography was obtained from parents and patients above 13 years of age. The patients were referred to the Department of Pediatric Surgery from the Department of Pediatric and Adolescent Endocrinology of the University Children’s Hospital of Krakow or regional centers.

### 2.1 Subject and the medical procedures

**Figure 1:**
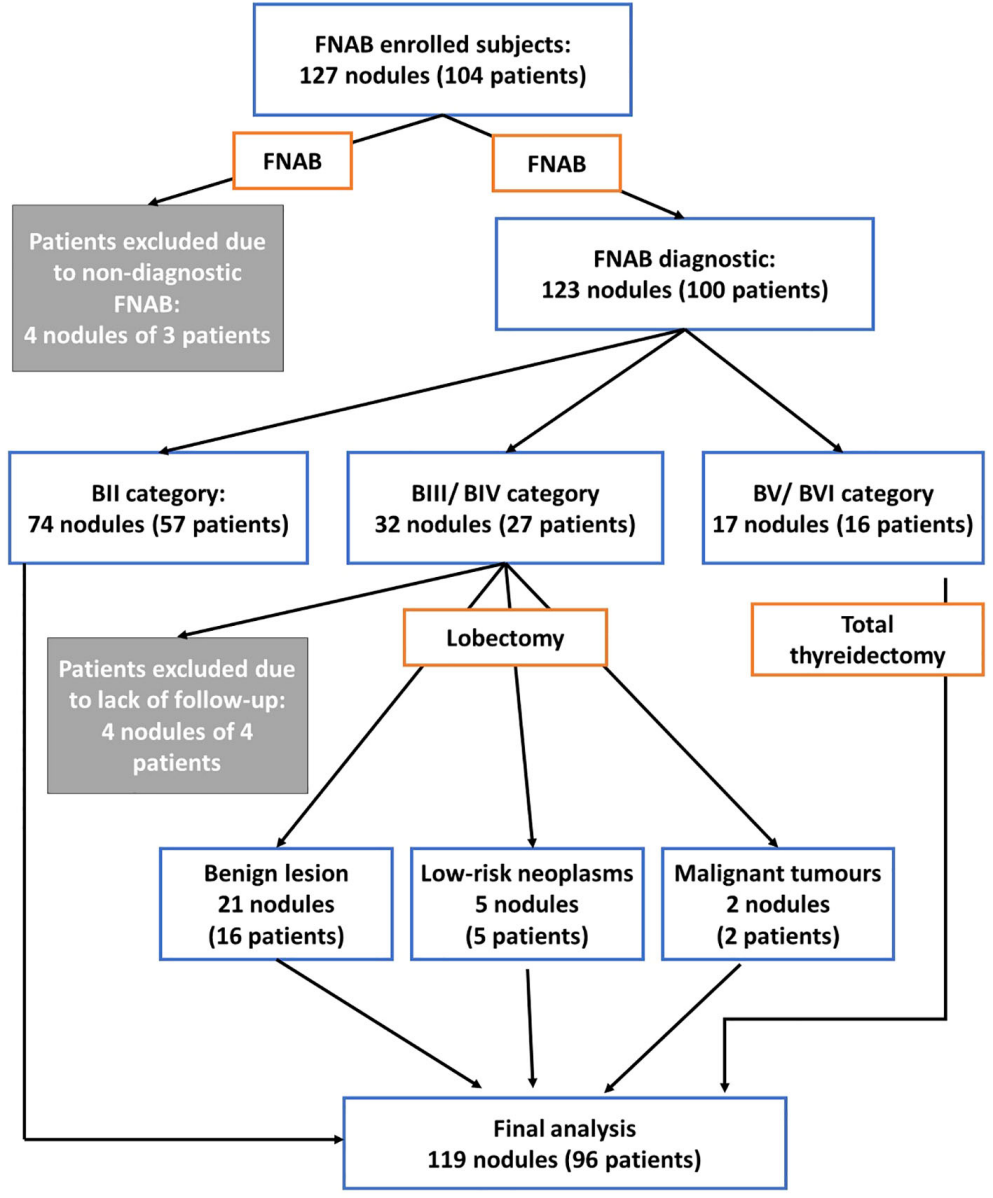
The workflow scheme of the study.

Elastography of 127 nodules was performed in 104 patients who were qualified for the FNAB procedure after a conventional ultrasound examination (**Figure 1**). In four nodules of three patients, FNAB was non-diagnostic. Each patient underwent elastography followed by FNAB. The result of elastography and the result of cytology (Bethesda scale) or histological examination were compared if the patient was qualified for surgical treatment. The surgical treatment included the removal of a lobe or the entire thyroid gland. The surgery was performed due to BIII–VI, tumor size >4 cm, and compression symptoms, according to the Polish and European recommendations (24). Four patients were excluded from further study. Their cytological result was BIII, but the result of histological examination was not known: in one case, the patient decided to undergo surgical treatment in a center for adults, another child was lost to follow-up, and two 18-year-old patients decided to continue observation.

**Figure 2:**
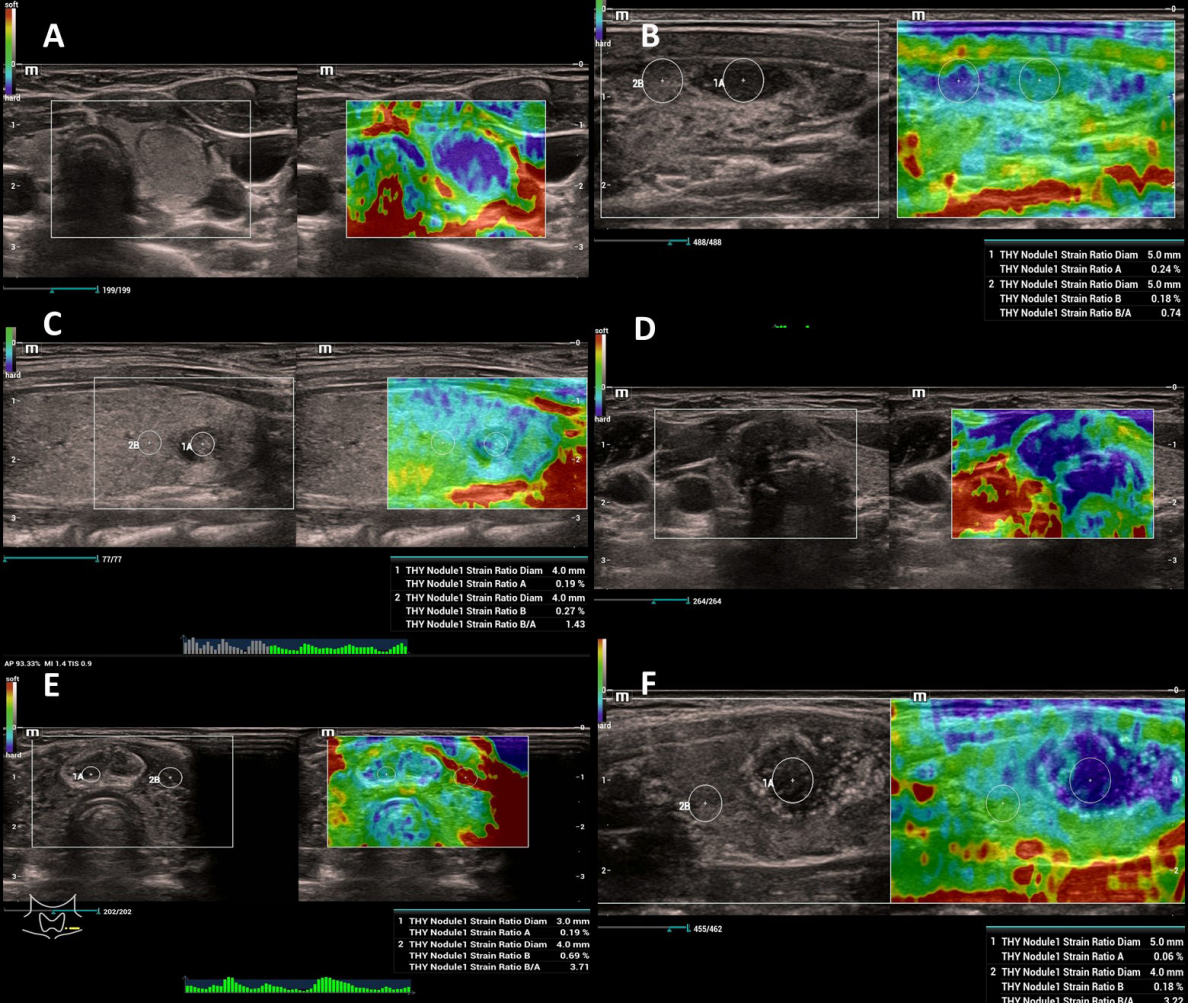
**(A)** A hyperplastic nodule of the left lobe of the thyroid gland, SR 2. **(B)** A benign nodule of the thyroid. Hashimoto disease; SR of nodule 0.74. **(C)** Follicular adenoma of the thyroid; SR 1.43. **(D)** Papillary carcinoma; SR 8.3. **(E)** Papillary carcinoma of the isthmus; SR 3.7. **(F)** Papillary carcinoma; SR 3.22. SR, strain ratio.

The ultrasonographic investigation was conducted by the same pediatric surgeon (AKW) certified in ultrasonography with more than 12 years of experience in pediatric ultrasonography. Several SR determinations (4-6) were performed for each nodule, in large-sized tumors from different regions of the nodule, and the mean value for the lesion was calculated. The ultrasound examination time of each patient was extended by 3–5 minutes. For this study, we used the Hitachi Aloka Arietta V 70 ultrasound scanner, a linear probe (L14-3Ws), and a hockey stick probe (L16-4Hs). The thyroid nodules were assessed in the B-mode option, color Doppler scales, and strain elastography. The SR index was obtained for each nodule (**Figures 2A–F**). Solid nodules or solid elements in solid-cystic tumors were studied. In a few cases, tumors filling the entire lobe of the thyroid gland were calculated as the ratio of muscle tissue to the thyroid nodule. FNAB was performed under local anesthesia with the use of a lidocaine cream applied on the skin above the lesion selected in ultrasound. In exceptional cases, the FNA biopsies were performed under general anesthesia, usually in young, uncooperative patients. The biopsy specimens were collected from one to four nodules of a given patient. The cytological sample from FNAB was passed directly to the pathologist participating in the procedure, fixed with 98% ethanol, and stained with hematoxylin–eosin (H&E). The cytological sample was assessed using the Bethesda scale by two pathologists; in selected cases, the material was sent for consultation to the reference center.

The postsurgical thyroid sample was sent to the Department of Pathology and fixed with 10% buffered formalin, the entire tissue sample was processed to obtain paraffin blocks, and the H&E slides were further examined according to the WHO and American Joint Committee on Cancer (AJCC) guidelines by pathologists (25). Based on histology, the 100 patients were divided into three groups: 1) benign lesions, 2) low-risk neoplasms, and 3) malignant neoplasms. The 123 nodules were biopsied from 100 patients. In four cases, the patient was biopsied again after 6–12 months, and the results were considered “new” in this study.

### 2.2 Data analysis

The descriptive data of the characteristics of the subjects were summarized using the statistical tools of Microsoft Excel. The data were presented as mean ± SD and median (with range and quintiles). The statistical analysis (tests of hypotheses) was performed using STATISTICA ver.13.3. A *p*-value of <0.05 was considered significant. Descriptive statistics of the study group (age of the respondents by gender) are shown in **Table 1**. The used tests were a) the Kruskal–Wallis test to verify the relationship between elastography parameters (continuous data) and the nodule malignancy (categories) and b) for the combined groups (benign and malignant nodules), the Mann–Whitney U test was employed to verify the continuous data.

**Table 1:** Descriptive statistics of the study group—age of the respondents by gender.

Age	Mean	SD	Min.	1st quartile	Median	3rd quartile	Max.
**Female (n = 79)**	16.1	2.6	7	15	16	18	20
**Male (n = 17)**	14.9	2.3	10	13	15	17	18
**Total (n = 96)**	15.9	2.5	7	15	16	18	20

**Figure 3:**
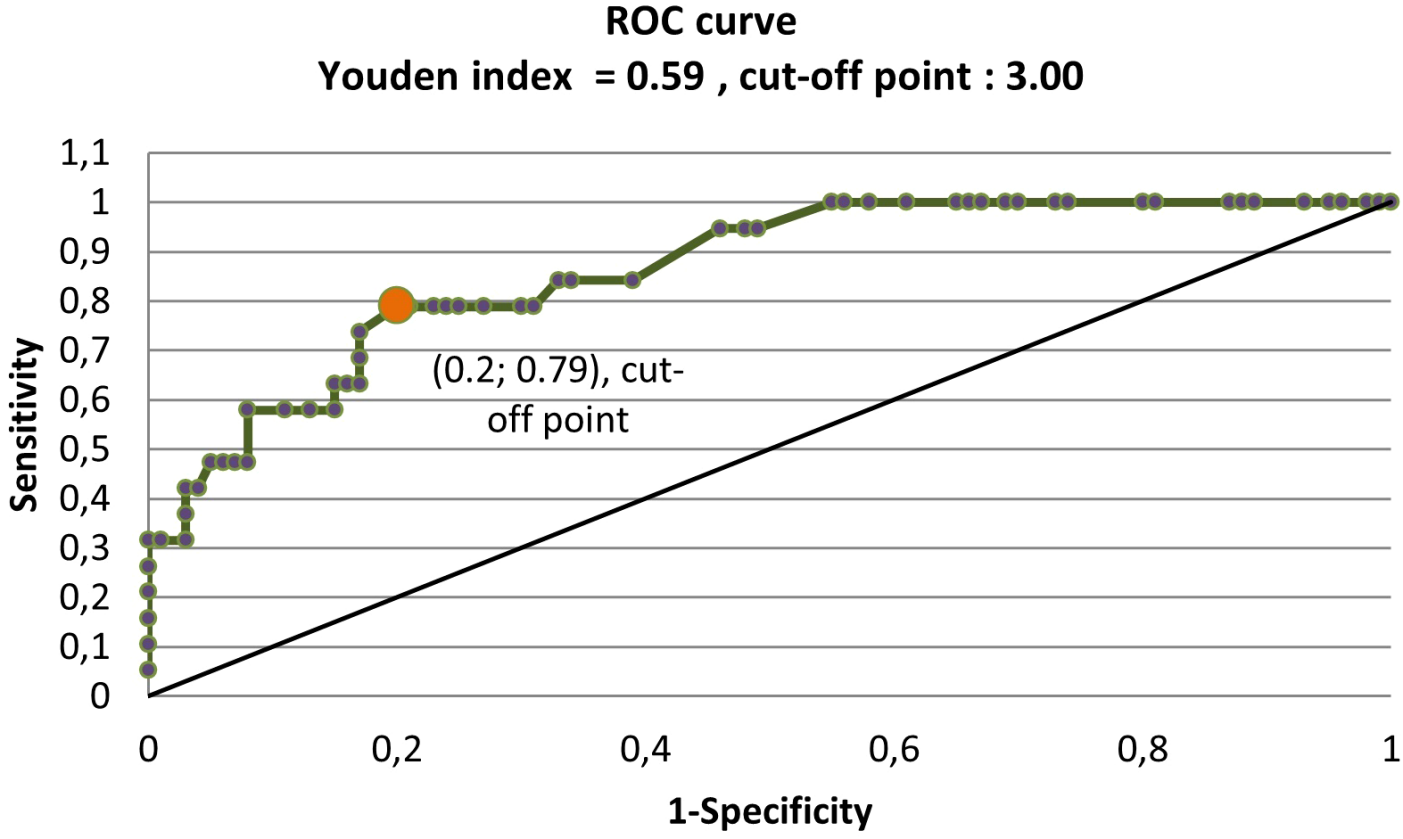
The ROC curve and Youden’s index. ROC, receiver operating characteristic.

To assess whether elastography may have a promising diagnostic value, the elastography and histopathology results were arranged in descending order according to the elastography values, and each verse was treated as a potential cut-off point on the receiver operating characteristic (ROC) curve. The authors calculated the sensitivity, specificity, and other parameters, such as the positive predictive value (PPV), negative predictive value (NPV), accuracy, false-positive ratio, false-negative ratio, likelihood ratio, error rate, max Youden’s index, and mean and median SR value of elastography in particular groups of nodules; the results are presented in **Tables 2**–**4**. The ROC curve (sensitivity, 1 − specificity) was plotted. Then, the value with the maximum Youden’s index (the distance of the point from the line slope of the tangent to the ROC curve, where y = x) was selected as the cut-off point. Conceptually, the maximum Youden’s index is the minimum distance between the upper left corner of a square with side 1 (i.e., the place where sensitivity and 1 − specificity can be the highest) and the point of the ROC curve (**Figure 3**).

**Table 2:** Mean SR value of elastography in histology-related groups.

	Mean	SD	Min.	1st quartile	Median	3rd quartile	Max.
Benign tumors	2.1	1.5	0.51	1.1	1.7	2.3	8
Low-risk neoplasms	2.5	0.9	1.9	1.9	1.9	2.9	3.9
Malignant tumors	5.6	3.3	1.7	3.1	4.7	8.5	11.7

SR, strain ratio.

**Table 3:** Descriptive statistics of SR indices in three groups of nodules.

Dependent variable: elastography	*p*-Value for multiple (double-sided) comparisons		
	Benign tumors	Malignant tumors	Unspecified tumors
Benign tumors		***p* < 0.001**	0.70
Malignant tumors	***p* < 0.001**		0.48
Unspecified tumors	0.70	0.48	

SR, strain ratio.

Values in bold denote statistical significance.

**Table 4:** The number of benign (combined group: benign and low-risk tumors) and malignant tumors with the mean, min, max, and median SR in each group.

	Number of tumors (%)	Mean	SD	Median	Min.	Max.
**Benign**	100 (84%)	2.16	149	1.7	0.51	8.0
**Malignant**	19 (16%)	5.68	3.3	4.7	1.70	11.7
**Total**	119 (100%)	2.71	2.27	1.9	0.51	11.7

SR, strain ratio.

## 3 Results

### 3.1 Subject

**Figure 4:**
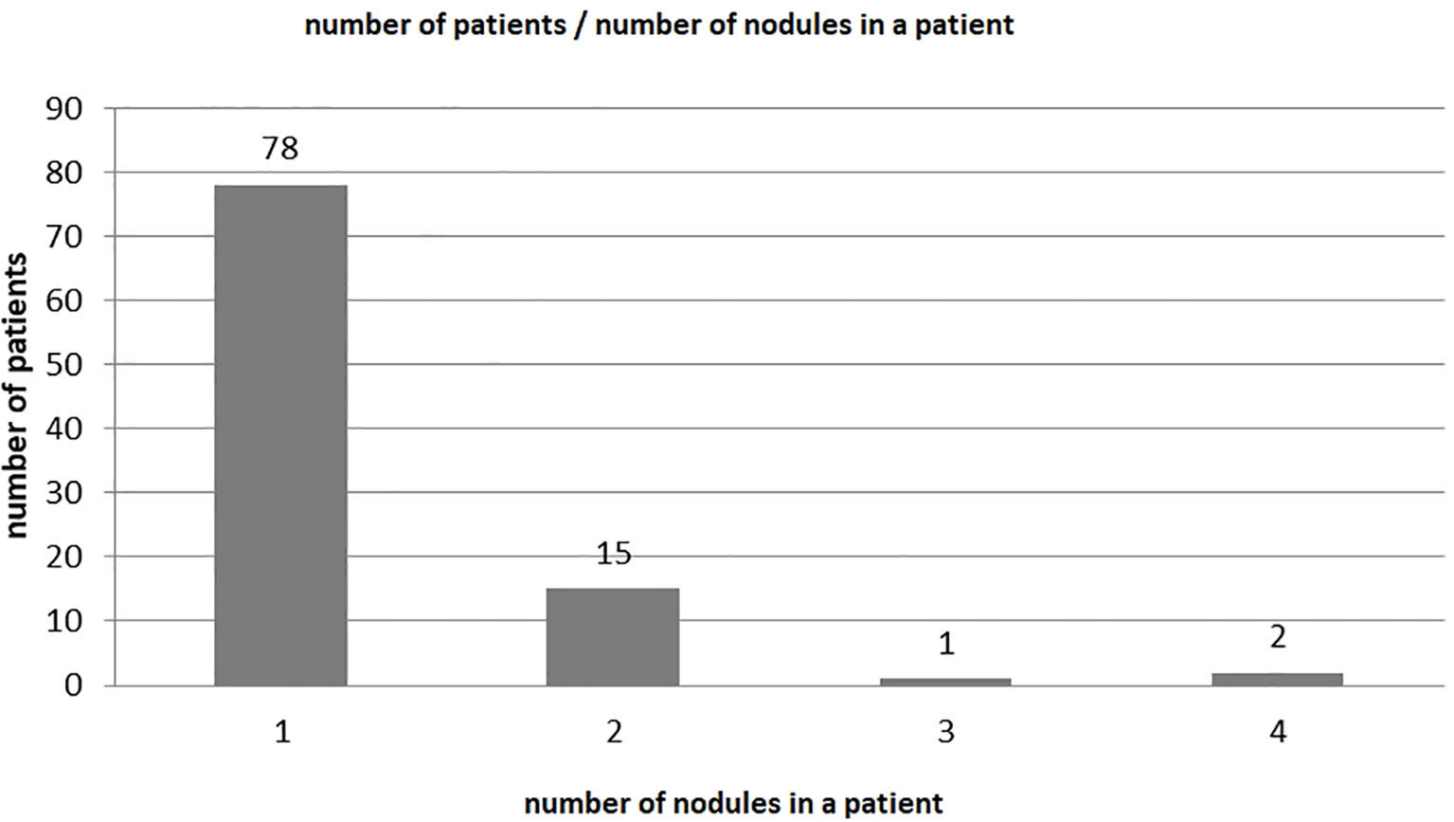
The number of nodules biopsied.

The final analysis involved 119 nodules of 96 patients. The 17 boys and 79 girls constituted 18% and 82% of the study group, respectively. The mean age was 16.1 years and 14.9 years for girls and boys, respectively (**Table 1**). Seventy-eight patients had a singular nodule, 15 patients had two nodules, one patient had three nodules, and two patients had four nodules (**Figure 4**). Hashimoto’s goiter was present in 24% of the analyzed group. The histology-based groups were different in size.

### 3.2 Pathological examination

The 119 nodules were assessed using the Bethesda scale, and 49 were histologically examined. n = 19 (16%) of nodules were malignant, and n = 5 (4%) were low-risk neoplasms (i.e., WDT-UMP, two nodules in two patients; FT-UMP, two nodules in one patient; and NIFTP, one nodule in one patient), and a total of 95 nodules (80%) were benign (21 resected and 74 biopsied with BII and observation). Among the malignant tumors, PTC prevailed: 17 nodules (89.5%) in 17 patients and two nodules representing medullary carcinomas (10.5%) from one patient were detected. Hashimoto’s goiter was present in 24% of the children, and in the Hashimoto patients, 17.4% had malignant lesions.

### 3.3 Elastographical findings

The SR rate in the group of malignant thyroid nodules was significantly higher (mean = 5.6, median = 4.7) as compared to that in the benign nodule group (mean = 2.1, median = 1.7).

**Figure 5:**
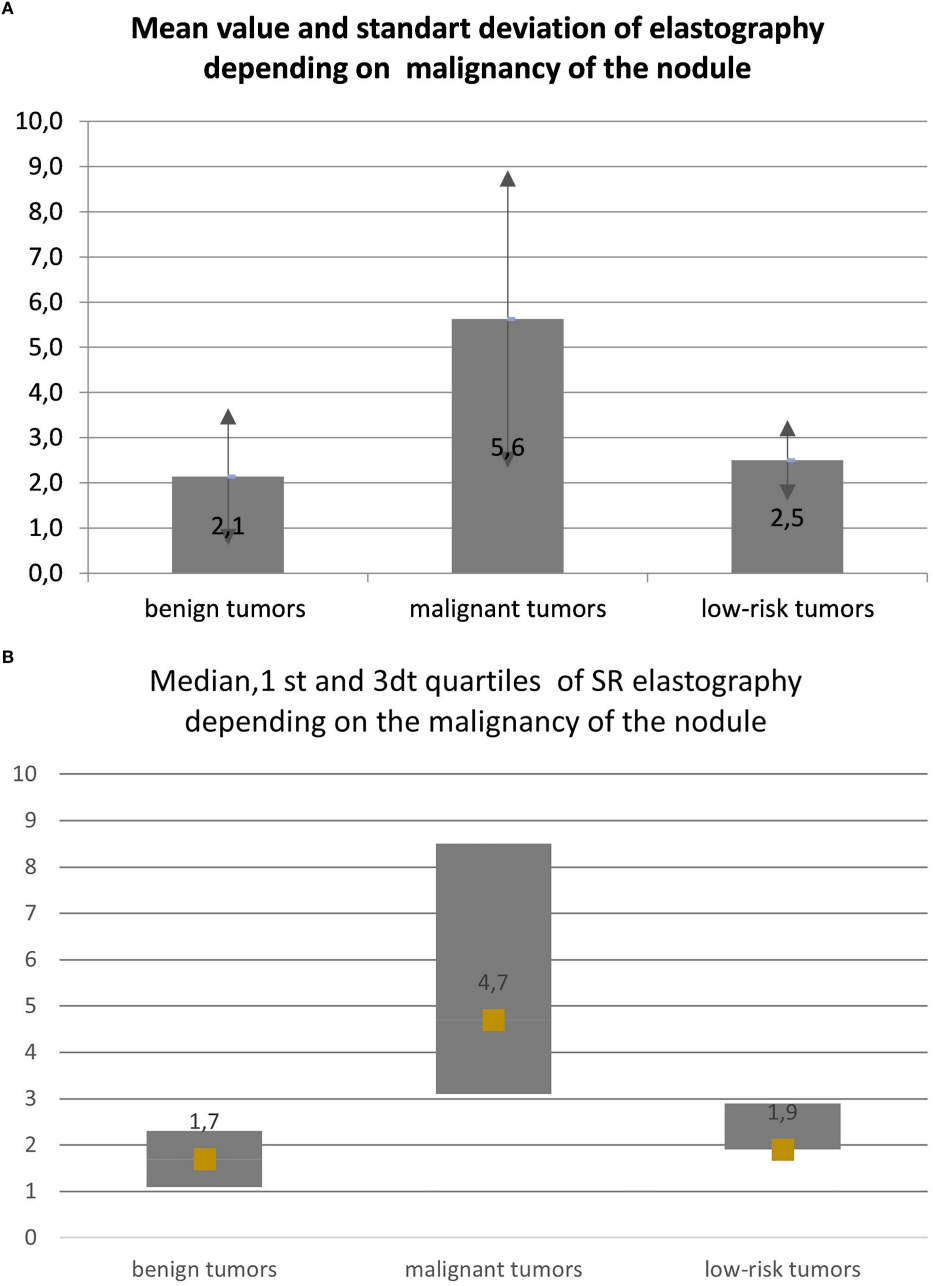
**(A)** The mean value and standard deviation of SR elastography depend on the malignancy of the nodule. **(B)** Median, first, and third quartiles of SR elastography depending on the malignancy of the nodule. SR, strain ratio.

The mean and median SR values in low-risk tumors were 2.5 and 1.9, respectively (**Table 2**, **Figures 5A, B**).

Median, first, and third quartiles of SR elastography depending on the malignancy of the nodule were 1.7 for benign tumors, 1.9 for low-risk neoplasms, and 4.7 for malignant tumors (**Table 2**, **Figure 5B**).

In the analyzed group, Hashimoto’s goiter was present in 24% of the children (N = 23) and malignant lesions in 17.4% of them (N = 4). The mean SR in benign nodules was 2.5, and the mean SR in malignant nodules was 5.6, but the groups were too small for statistical significance.

Then, a test for multiple comparisons was performed to check which groups revealed statistically significant differences. The results are presented in **Table 3**.

It was found that there was a significant difference in the elastography values between malignant and benign tumors and between malignant and low-risk tumors. We did not find any statistically significant differences between benign lesions and low-risk tumors. Since there was no statistically significant difference in the level of elastography between benign lesions and tumors with an uncertain malignant potential, both groups were combined to form a group of benign tumors (benign + unspecified malignancy) and a group of malignant tumors. The mean SR in benign nodules was 2.16. The mean SR in malignant nodules was 5.67. The results are presented in **Table 4**.

The basic parameters with such a cut-off value were as follows: SR = 3, PPV = 0.429, NPV = 0.952, sensitivity = 79%, and specificity = 80% (**Table 5**).

**Table 5:** The basic parameters and their cut-off values.

SR	True positives	False positives	False negatives	True negatives	Sensitivity	Specificity	1 − Specificity	Accuracy
3	15	20	4	80	0.789	0.800	0.200	0.798
SR	PPV	NPV	FPR	FNR	(+) - LR(+)	Error rate	Youden	Likelihood ratio (-) -LR(−)
3	0.429	0.952	0.200	0.211	3.947	0.202	0.589	0.263

SR, strain ratio value; PPV, positive predictive value; NPV, negative predictive value; FPR, false-positive ratio; FNR, false-negative ratio; LR, likelihood ratio.

## 4 Discussion

### 4.1 General discussion

Thyroid nodules in children are characterized by a twofold to threefold increased risk of being malignant when compared to those in the adult population (14). Various scales are being sought by pediatric surgeons to select nodules for invasive diagnostic procedures. Ultrasound elastography has shown promising results for the non-invasive assessment of the thyroid gland/nodule.

Strain elastography allows for obtaining knowledge about nodules that are not palpable, and in the era of universal availability of ultrasound examinations, it is extremely important that even small nodules—5–10 mm deep in the thyroid parenchyma—can be well and quickly diagnosed or observed. Refined test schemes allow for making an accurate diagnosis without exposing patients to unnecessary invasive diagnostic management and procedures that, in the group of pediatric patients, involve general anesthesia.

In the literature, strain elastography is mainly studied in the adult patient population. In the work by Wang et al., the SR and the elastography score system were compared—the results seem to be more favorable for the assessment according to the SR index, with a cut-off point of 3.855 for malignant lesions (26). Also, Cantisantis et al. (2013) presented the assessment of thyroid nodules using conventional ultrasound and elastography methods in adult patients before thyroidectomy. They analyzed 147 nodules in 123 patients who were qualified for surgical treatment due to nodular goiter or single thyroid nodules. The sensitivity and specificity of the ultrasonography (US) score were approximately 56% and 72%, respectively, whereas those of the strain ratio were 93% and 89%. The cut-off point for malignant nodules that were >2 was determined using ROC curve analysis. The elastosonography was more accurate than the B-mode ultrasonography and color Doppler ultrasonography in characterizing thyroid nodules (27).

In another study, the assessment of SR, shear wave elastography (SWE), and conventional ultrasound features were compared. SR was demonstrated to be characterized by a better performance than the TI-RADS classification (28).SE yielded the highest performance with a sensitivity of 82.7% and specificity of 92.7% with an area under the ROC (AUROC) of 0.877, PPV of 75.4%, and NPV of 95.2. The SE evaluation showed the best diagnostic accuracy compared to the SWE (expressed in kPa) (p < 0.05) and to TI-RADS.

In the large meta-analysis by Sun et al. (2014), 1,063 nodules in 983 patients for the strain ratio studies were analyzed. The overall mean sensitivity and specificity of ultrasound elastography for the differentiation of thyroid nodules were 0.85% and 0.80 for the strain ratio assessment, respectively; the area under the curve for the strain ratio amounted to 0.9285 (29). In the present study, the specificity was the same (80%), but the sensitivity was lower (79%).

In the study by Yang et al. (2020), the authors underlined the importance of SE in detecting papillary carcinoma due to its stiffness, which is noted in a vast majority of children (30).

In the present study, papillary carcinoma was detected in almost 90% of all the histological results of malignant lesions.

There are not many studies on thyroid nodule elastography in pediatric patients (31, 32). The study by Cunha et al. (2019) presented the assessment of the elastography scoring system, based on a color scale in a small group of 32 patients (31). Currently, there is no necessity to rely only on a subjective analysis of the color map, as more precise methods of evaluation of elastography such as SE or SWE are available.

Children with Hashimoto’s disease are diagnosed at an earlier stage of cancer, but due to the heterogeneity of the thyroid parenchyma altered by the autoimmune process, ultrasound evaluation of the B-mode is difficult. The elastography helps to pinpoint the most suspicious areas. Strain elastography in pediatric patients was assessed in the Borysewicz-Sańczyk et al. (2016) study; the authors proved that autoimmune thyroid disease did not limit the use of SE in the assessment of thyroid nodules (33). The SR of the nodules was higher in malignant as compared to benign nodules. Two large groups of patients were compared: with autoimmune thyroid disease (AITD) and without AITD. The SR of benign nodules in the group of patients with AITD was lower compared to that of the group of patients without AITD (AITD 2.92 ± 1.89, non-AITD 3.67 ± 2.62), but the group of thyroid cancers accounted for only 2% in both groups, and their assessment was not statistically significant (33).

In another paper, Borysewicz-Sańczyk et al. (2022) evaluated 17 nodules for ultrasound features in grayscale and elastography. The strain ratio in the group of thyroid nodules diagnosed as malignant was significantly higher than that in benign nodules (6.07 vs. 3.09, p = 0.036), with only five nodules being malignant (34).

When it comes to shear wave elastography in the assessment of thyroid nodules in adult patients, there are few reports available, the results of which are inconclusive and require further investigation (35). In the pediatric population, research on the evaluation of shear wave thyroid nodules is very limited. In only one study to date, Hazem et al. (2021) evaluated 72 thyroid nodules in children for SWE and final histology with promising results—42.2 kPa was the best cut-off value between benign and malignant lesions (36). It seems that this may be a complementary method to SE. Also in a large meta-analysis, Hu et al. (2017) showed by analyzing strain elastography in adults and SWE that the specificity of SE was statistically higher than that of SWE, which suggests that compared with SWE, real-time elastography (RTE) may be more accurate in differentiating benign and malignant thyroid nodules (37).

In our study, malignant lesions constituted 16% of all nodules, a finding that was statistically significant. Within the analyzed cohort, Hashimoto’s thyroiditis was present in 24% of cases, and malignant lesions were identified in 19% of them. The mean strain ratio (SR) for benign nodules was 2.5, while for malignant nodules, it was 5.6. Although there was a significant difference in mean SR values between benign and malignant nodules, overlapping values existed, which precluded the absolute exclusion of malignancy. Moreover, the sensitivity and specificity of elastography were insufficient to recommend its routine application in the evaluation of TI-RADS scores.

The four false-negative cases included two papillary carcinomas in small nodules (approximately 5 mm), one medullary carcinoma, and one papillary carcinoma in a nodule measuring 16 × 14 × 13 mm. In these cases, elastography did not provide useful diagnostic information.

The strength of this study lies in its correlation of elastography results with final histological findings, with a particular focus on pediatric thyroid surgery, a rapidly advancing field. Current European guidelines for the management of pediatric thyroid nodules recommend observation of lesions classified as Bethesda category III in adolescents due to their associated malignancy risk (38). Elastography outcomes may help guide surgical decision-making by supporting or delaying the timing of intervention (39, 40).

### 4.2 Study limitations

The size of the nodule was not analyzed, and it seems that it may be relevant to the outcome of the elastography; a focus on cancerous nodules smaller than 5 mm may not affect the stiffness of a nodule of a larger size, or cancerous nodules larger than 20 mm may be missed. Due to the more aggressive course of thyroid cancer in children, additional features of the nodule are still being sought, which will entitle us to perform FNAB and surgical treatment or allow for further observations of the lesion.

## 5 Conclusions

Elastography demonstrated significant value in differentiating between benign and malignant nodules, with a cut-off point of >3 identified in this study. However, there are limitations to its use, particularly in small thyroid nodules, as highlighted in the findings. Strain elastography (SE) may serve as an adjunctive tool for diagnosing suspicious thyroid nodules in pediatric patients, but further research through multicenter studies is necessary to improve its efficacy in the pediatric population.

## Data Availability

The raw data supporting the conclusions of this article will be made available by the authors, without undue reservation.
